# Stress mechanism involved in the progression of alcoholic liver disease and the therapeutic efficacy of nanoparticles

**DOI:** 10.3389/fimmu.2023.1205821

**Published:** 2023-09-29

**Authors:** Hiral Aghara, Prashsti Chadha, Devangi Zala, Palash Mandal

**Affiliations:** P D Patel Institute of Applied Sciences, Charotar University of Science and Technology, Anand, Gujarat, India

**Keywords:** alcoholic liver disease, metabolism, gut-liver, stress mechanism, nanoparticle mediated treatment

## Abstract

Alcoholic liver disease (ALD) poses a significant threat to human health, with excessive alcohol intake disrupting the immunotolerant environment of the liver and initiating a cascade of pathological events. This progressive disease unfolds through fat deposition, proinflammatory cytokine upregulation, activation of hepatic stellate cells, and eventual development of end-stage liver disease, known as hepatocellular carcinoma (HCC). ALD is intricately intertwined with stress mechanisms such as oxidative stress mediated by reactive oxygen species, endoplasmic reticulum stress, and alcohol-induced gut dysbiosis, culminating in increased inflammation. While the initial stages of ALD can be reversible with diligent care and abstinence, further progression necessitates alternative treatment approaches. Herbal medicines have shown promise, albeit limited by their poor water solubility and subsequent lack of extensive exploration. Consequently, researchers have embarked on a quest to overcome these challenges by delving into the potential of nanoparticle-mediated therapy. Nanoparticle-based treatments are being explored for liver diseases that share similar mechanisms with alcoholic liver disease. It underscores the potential of these innovative approaches to counteract the complex pathogenesis of ALD, providing new avenues for therapeutic intervention. Nevertheless, further investigations are imperative to fully unravel the therapeutic potential and unlock the promise of nanoparticle-mediated therapy specifically tailored for ALD treatment.

## 1 Introduction

The liver, as one of the largest immune organs in the human body, plays a crucial role in maintaining overall immunity by creating an immunotolerogenic environment (1–3). Comprised mainly of hepatocytes, which make up 70% of its composition, the liver also contains various non-parenchymal cells such as hepatic sinusoidal endothelial cells, hepatic stellate cells (HSC), Kupffer cells (liver macrophages), and pit cells (liver-specific natural killer cells) (4). The parenchymal cells of the liver produce innate immunity proteins, bactericidal proteins, and opsonins, contributing significantly to the body’s innate immune response (5, 6). Additionally, the liver has unique characteristics where liver allografts are more readily accepted due to heightened innate immunity and suppressed adaptive immunity compared to other organs (5, 7). However, if the liver’s immunotolerant environment is disrupted by factors such as viral infections, an unhealthy diet, or certain drugs, it can lead to liver disease.

Excessive and prolonged alcohol intake is a major cause of liver disease, specifically known as alcoholic liver disease (ALD). ALD, along with non-alcoholic liver disease (NAFLD), which is now referred to as metabolism-associated liver disease, pose significant health problems worldwide. Alcohol abuse is considered the leading risk factor for disease and disability globally, contributing to a mortality rate of 4.8% worldwide, with approximately 10% of these mortalities occurring in India (8). Furthermore, alcohol’s negative impact extends to socioeconomic activities. To provide context, a standard beer or wine cooler typically contains 5% alcohol, with 355mL of the drink containing 14 grams of pure alcohol. Malt liquor contains around 7% alcohol, with 355mL of the drink containing 19.60 grams of pure alcohol. Furthermore, 740mL of 12-14% wine contains approximately 70-80 grams of pure alcohol. The dietary guidelines in the United States suggest that consuming one drink per day for women and two drinks per day for men is considered moderate and does not typically lead to liver disease (9).

**Figure 1:**
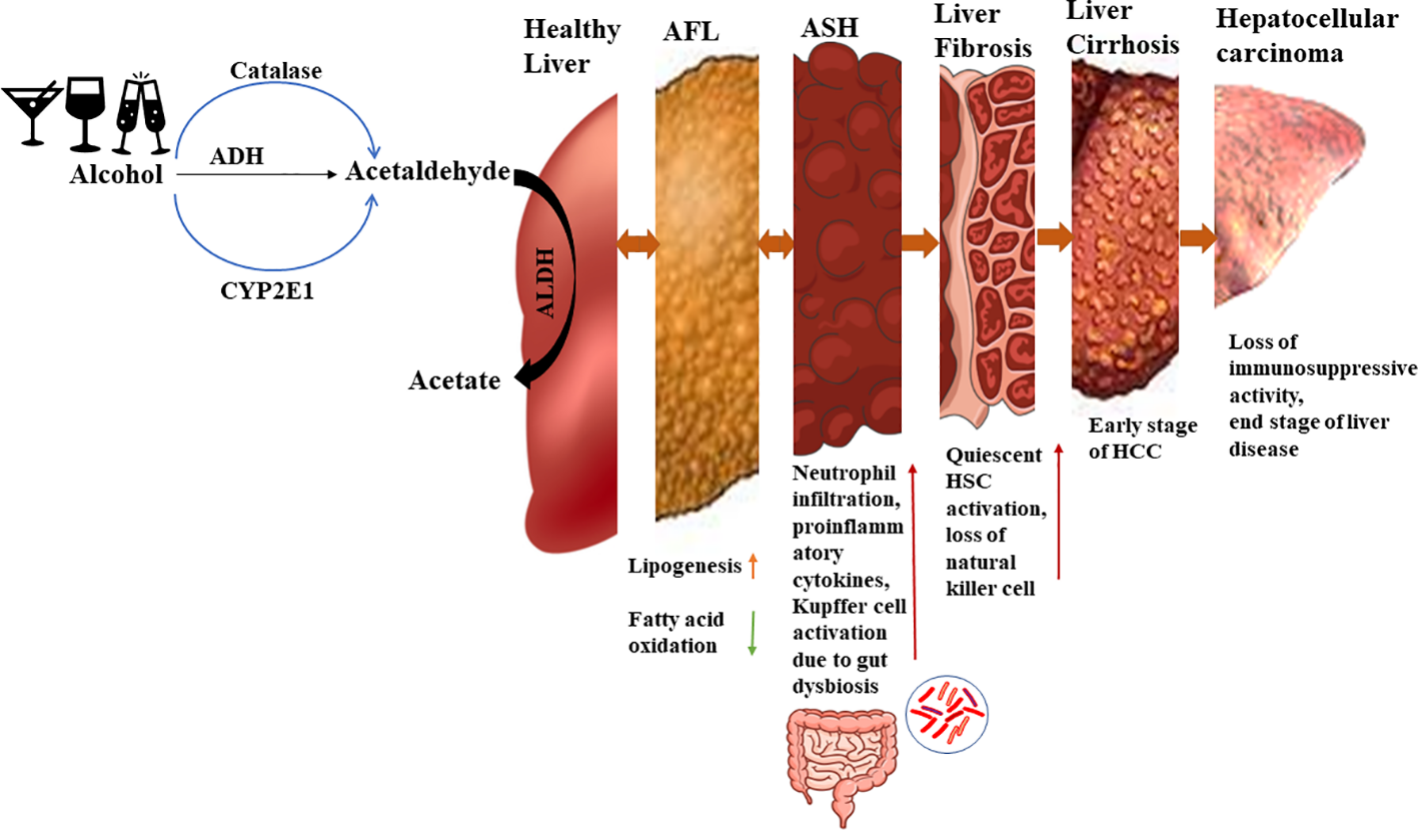
Alcohol metabolism and ALD progression. In a healthy state, ethanol is converted to acetaldehyde by alcohol dehydrogenase and then to acetate by aldehyde dehydrogenase. But in presence of excess ethanol, the MEOS pathway is activated leading to increased acetaldehyde levels. During the initial stage of alcoholic liver disease (ALD), there is an increase in lipid generation and reduction in fatty acid oxidation, resulting in lipid accumulation in the liver. Approximately 10-35% of individuals progress to alcoholic steatohepatitis, characterized by sterile inflammation and elevated cytokines and chemokines. Kupffer cells are activated by gut endotoxins during this stage. Continued heavy alcohol consumption leads to liver fibrosis, where around 40-50% of individuals experiencing activation of quiescent hepatic stellate cells due to Kupffer cell activation. Natural killer cell activity declines, further promoting hepatic stellate cell activation which accelerates fibrosis. Subsequently, 8-20% of individuals develop liver cirrhosis, an early stage of end-stage liver disease, and approximately 3-10% progress to hepatocellular carcinoma, an advanced stage of liver disease.

The pattern of ALD progression is illustrated in **Figure 1** (10). If an individual consumes more than 40g of pure alcohol per day for an extended period, they may develop alcoholic fatty liver disease (AFL). In AFL condition, excess alcohol would interrupt fat oxidation by inhibiting β oxidation of fatty acid and 5ʹ-AMP-activated protein kinase (AMPK). If alcohol consumption is halted at this stage, AFL can be reversed to a healthy state. However, if an individual continues to consume high amounts of alcohol, inflammation will occur along with fat deposition, leading to the stage called alcoholic steatohepatitis (ASH). At this stage gut dysbiosis also takes place and it interacts with liver residential macrophages which further activate quiescent hepatic stellate cells. It will produce collagen and extra cellular matrix protein. Some growth factors like TGF-β and platelet derived growth factor- β are most known for HSC proliferation. Due to quiescent Hepatic Stellate Cells (HSCs) activation, the disease progresses to liver fibrosis and at this stage retinol storage decreases and extracellular matrix increases. Activated quiescent HSCs produce more collagen which would further promote capillarization of hepatic sinusoidal endothelial cells and interrupt nutrient transport. Further, the disease progresses to alcoholic cirrhosis and hepatocellular carcinoma, which denote end-stage liver disease (11, 12). 

## 2 Alcohol metabolism

Alcohol metabolism occurs via two main pathways, the alcohol dehydrogenase (ADH) pathway and the non-ADH pathway which is also recognized as the microsomal ethanol oxidizing system (MEOS) (13). The ADH pathway is a primary pathway for ethanol metabolism in liver cells and occurs in cytosol due to presence of necessary enzymes. The MEOS pathway involves enzymes like catalase and CYP2E1 which are present in the peroxisomes and microsomes of cells. Pathways involving all the above enzymes are oxidative pathways. The non-oxidative metabolism of ethanol is not prominent but leads to the formation of fatty acid ethyl esters (FAEEs) or phosphatidic acid (PA) (14). The literature reports that if the alcohol concentration in blood is less than 10M/L, then alcohol metabolism will take place via the ADH pathway (15). As the blood alcohol concentration elevates, the other two pathway will also participate in ethanol metabolism.

Alcohol catabolism occurs, and initially acetaldehyde is formed and further converted into acetate via acetaldehyde dehydrogenase (ALDH). Since acetaldehyde is a toxic compound and acts as a mutagen, it is rapidly catabolized to acetate (16). However, as the alcohol concentration increases, more acetaldehyde is formed, posing a risk to cells. Acetaldehyde can bind to proteins and DNA, leading to structural and functional changes, the generation of neoantigens, and activation of the immune system (17). The progression of ethanol metabolism is accompanied by an increase in reactive oxygen species (ROS). These ROS contribute to the formation of lipid peroxides, which can further deform the structure of DNA and proteins through binding (18). Excessive lipid peroxidation results in the formation of electrophile products like Malondialdehyde (MDA) and 4 Hydroxynonenal (4-HNE), which bind with essential proteins, disrupting cellular homeostasis (18, 19). **Figure 1** shows alcohol metabolism and ALD progression. 

With an increase in alcohol intake, the cellular antioxidant defense mechanism weakens. Antioxidants such as glutathione and superoxide dismutase (SOD) are naturally present in cells, and their levels decrease as alcohol consumption rises. This results in an inverse relationship between the concentration of free radicals and antioxidants within the cells. Furthermore, excessive alcohol intake can also have an impact on vitamin levels. The elevated alcohol concentration triggers various stress mechanisms within the body.

## 3 Alcohol and oxidative stress

**Figure 2:**
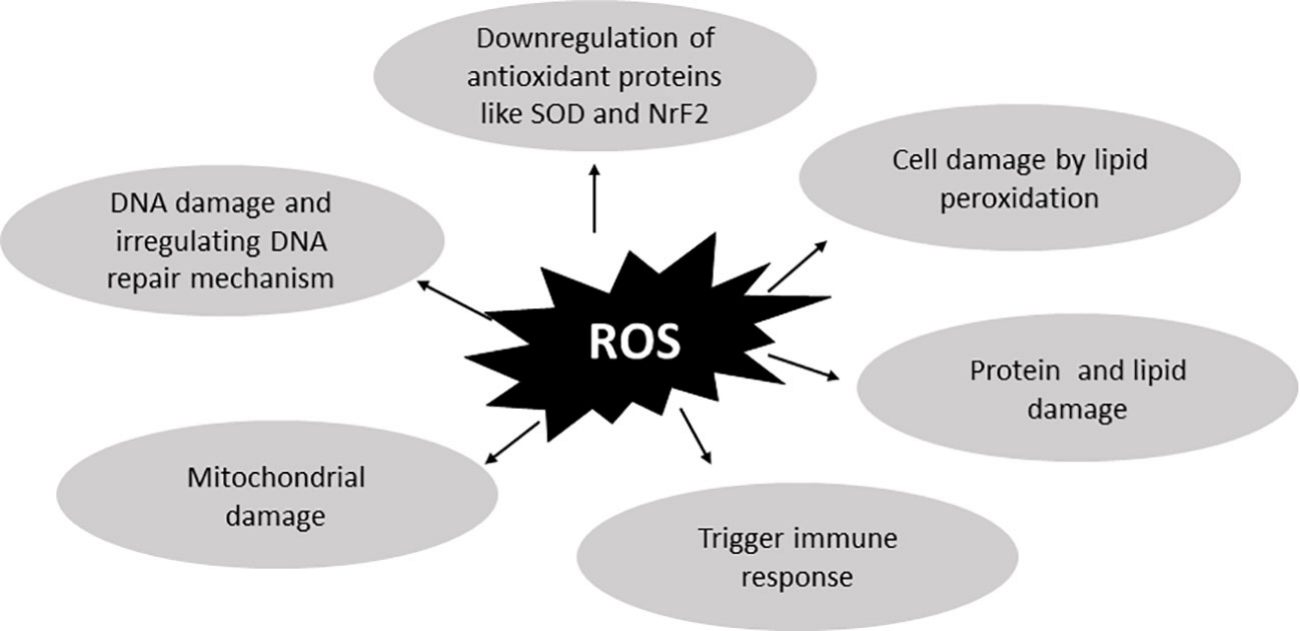
ROS-related damage to cells. Reactive oxygen species are important for cell-to-cell crosstalk but in excessive amounts they lead to diseased conditions. By binding protein, lipid, and DNA, they damage the cellular mechanism and also dysregulate antioxidant activities. They also dysregulate the DNA repair mechanism and cause more DNA damage. Further increased ROS concentrations damage cells by generating lipid peroxidation which further damages cell membrane and causes cell death.

A proper balance between antioxidants and reactive oxygen species (ROS) is generally maintained in every cell (20). ROS, being highly unstable free radicals, are generated by cellular mechanisms and play essential roles in cellular communication and other processes. Various species of ROS, including H_2_O_2_, O_2_^-^, and `OH are produced due to increased NOX4 activity (21). While ROS have several detrimental effects, they are generally important for transmitting messages and eliciting responses. There are two types of antioxidant mechanisms that remove excess ROS (22, 23) and both are equally vital for cellular function. Maintaining homeostasis between these two mechanisms is crucial. Imbalances in these mechanisms can lead to disease conditions (24, 25) which may arise from factors such as lifestyle choices, drug use, chemotherapy, and other epigenetic factors. Within cells, two types of antioxidant mechanisms exist. The first is an endogenous or enzymatic antioxidant mechanism generated within cells, while the second is an exogenous or non-enzymatic antioxidant mechanism supplemented by food products (26). Elevated ROS levels can damage cells and activate apoptosis-related mechanisms. ROS also play significant roles in various cell signaling pathways, such as NFκB, MAPK, ion channeling, and the ubiquitin proteasome response (27). These pathways can further trigger Endoplasmic reticulum (ER) response and other pathways, which will be discussed later. **Figure 2** illustrates the damage caused by ROS to cells (28–32). 

Ethanol and acetaldehyde both are toxic to cells. The presence of these toxins results in mitochondrial damage further affecting various mechanisms which damage the cells (32). There are numerous factors involved in ROS generation. Therefore, it is important to understand these mechanisms and the harm caused by ROS generation in ALD.

### 3.1 Ways of ROS generation and disease progression in ALD

At the early stage of ALD, fat generation occurs and the oxidation rate of fatty acids is slower (33). Even at the first stage, there are many elements that help ROS generation. Some are mentioned below:

- NAD^+^: NADH ratio: As the conversion of ethanol to acetaldehyde and acetate occurs, it also generates NADH, which dysregulates the cellular redox potential (10). Numerous metabolic processes depend on NAD+ since it is essential for maintaining the cellular levels of sirtuins (34), which play a crucial role in the antioxidant system and DNA repair in cells (35, 36). Additionally, sirtuins are important for the brain in maintaining circadian rhythm (37). A reduced NAD+:NADH ratio impacts histone deacetylase activity (38). The increased NADH also affects the antioxidant defense mechanism by reducing its power (39).
- Sterol regulatory binding protein (SREBP1C) and other early growth responses (EGR1) are activated by the CYP2E1 cycle, which in turn activates the lipogenic gene (33, 40). According to (41–44), CYP2E1 is crucial in the detoxification of xenobiotics and other hazardous compounds. The detoxification of bioactive compounds generates toxic compounds or carcinogenic products which further contribute to disease progression (45, 46). In liver cells, ROS generation is significantly affected by the activity of CYP2E1 (47, 48).
- According to Vasiliou et al., (49), the microsomal ethanol-oxidizing system (MEOS) pathway plays a significant role in ethanol sensitivity. An increased acetaldehyde concentration further reduces the ability of ALDH2 to convert acetaldehyde to acetate (13, 50, 51). Acetaldehyde is a carcinogenic substance (52) that further alters many biomolecule components and produces ROS (16, 53). It also leads to the formation of DNA adducts, causing mutations, and induces changes in protein structure, resulting in the formation of neoantigens. These neoantigens are further detected by T cell immune cells and form inflammatory responses (17). In addition, acetaldehyde also interacts with other mechanisms (54–56).
- Excessive alcohol consumption damages the mitochondria, which leads to ROS production. An elevated amount of NADH can hinder ATP production and negatively impact the electron transport chain. Excessive alcohol consumption also decreases the function of mitochondrial respiratory complexes, specifically complex I, III, IV, and V, which are involved in converting ADP to ATP. This metabolic state promotes higher H2O2 production. Ethanol further amplifies the imbalance of oxidative phosphorylation and ROS generation (25, 57–59). According to (60), increased NADH levels contribute to more ROS formation, which can interact with cellular components such as lipids, proteins, nucleic acids, and especially mitochondrial DNA (mtDNA), enhancing oxidative stress and leading to apoptosis. ROS production affects mtDNA and compromises cellular energy metabolism, resulting in the production of even more ROS. In summary, higher concentrations of ROS damage mitochondria, leading to further elevated concentrations of ROS.
- Overall, in all the other mechanisms, there is one more mechanism which has been studied and is still being explored regarding ALD progression. It also damages mitochondria, produces more ROS, and contributes to disease progression. Necroptosis is a cell death mechanism which is activated due to RIPK1 and RIPK3 (receptor interacting protein kinase 1 and 3) (61, 62). As mitochondrial ROS generation increases, it also triggers inflammation. Damage associated molecular patterns (DAMP), interferon, death ligand-like tumor necrosis factor, Fas ligand, toll-like receptors, and Tumor necrosis factor-related apoptosis-inducing ligand can activate RIPK1 and RIPK3. As alcohol accumulation increases, it activates RIPK1 and RIPK3. Individually RIPK1 can cause inflammatory activity by neutrophil infiltration and producing proinflammatory cytokines and chemokines. Furthermore, RIPK3 is also elevated due to overexpression of cytochrome p450E1. As RIPK3 phosphorylates and it interacts with mixed lineage kinase domain-like protein (MLKL) and leads to membrane disruption and necroptosis (63, 64). By that mechanism is also increases oxidative stress by generating more ROS and inflammatory response (65). Further molecular mechanisms still need to be clarified to prevent the progression of the disease.

ROS not only affects main pathways but also hinders non-relevant pathways, such as Brahma-related gene 1 (BRG1) expression (31). BRG1 is responsible for the activation of various antioxidant mechanisms which are mutated due to excess ROS (66). Even the overexpression of BRG1 further correlates with SREBP1c and contributes to lipid metabolism (67–69). Research is still underway to explore the connection between BRG1 and other inflammatory genes and pathways, as there are conflicting findings in some studies regarding the formation and role of BRG1 (70). The relationship between BRG1 and other pathways needs to be explored in regard to ALD.

ROS and acetaldehyde have the ability to disrupt the structure of proteins. In the presence of intracellular stress factors such as the accumulation of misfolded proteins in the endoplasmic reticulum (ER), a chaperone-mediated stress response is activated to aid in the recovery and restoration of ER function. However, in cases where recovery is not feasible and the stress becomes overwhelming, the unfolded protein response (UPR) directs the cell towards either autophagy, which helps maintain cellular homeostasis, or apoptosis, leading to the clearance of the affected cell. Autophagy pathways, including PERK/ATF4, IRE1α, ATF6, and Ca+, can be activated and play a dual role. They can inhibit apoptosis by dampening the activity of apoptosis-associated caspases, thereby promoting cell recovery. Alternatively, they can also induce apoptosis, leading to the removal of accumulated proteins, lipid droplets, and damaged cellular components through autophagy (71). However, excessive alcohol accumulation can inhibit autophagy, which contributes to disease progression by generating endoplasmic reticulum stress (72).

## 4 Alcohol and ER stress

Cell damage can be caused not only by oxidative stress but also by ER stress, which acts as a secondary hit model for disease progression (73). The ER-associated degradation (ERAD) reaction is activated in response to unfolded or damaged proteins, aiming to maintain homeostasis. Under adverse conditions, ERAD supports the preservation of cellular equilibrium. Oxidative stress increases the production of ROS, which, in turn, triggers the activation of ER-associated genes due to stress metabolites, protein adducts, and protein misfolding. Additionally, other cellular responses, such as increased synthesis of CYP2E1 and a decreased liver S-adenosylmethionine (SAM)/S-adenosylhomocysteine (SAH) ratio, contribute to the development of ER stress (74, 75). SAM acts as a methyl donor and aids in the formation of glutathione, an important antioxidant. SAH, on the other hand, acts as a potential inhibitor of methylation. Furthermore, decreased NAD+ levels during alcohol metabolism directly increase the concentration of N-nicotinamide methyltransferase (NNMT), which plays a crucial role in maintaining the balance between NAD+ metabolism and the methionine cycle. Increased NNMT levels further elevate SAH. The decreased SAM : SAH ratio leads to an increase in CYP2E1, lipid peroxidation, the upregulation of ER stress marker genes, and prevention of DNA methylation (76–79). In ER stress, the activation of the PERK-ATF4 pathway plays a critical role in hepatic NNMT activation. Some researchers have reported that NNMT inhibition protects against alcohol-induced fatty liver disease and the generation of hepatic *de novo* lipogenesis (80, 81). There is a connection between oxidative stress and ER stress which still needs to be explored.

Various types of proteins are targeted in ALD. Excessive alcohol intake and its byproducts can cause denaturation or deformation of many proteins. As hepatocytes are rich in ER, they play a crucial role in maintaining protein homeostasis within cells. In adverse conditions, the unfolded protein response (UPR) becomes more prominent and helps maintain cellular homeostasis. However, prolonged activation of UPR can lead to inflammation and interaction with other pathways (82). The UPR consists of three main branches that increase the folding capacity of the ER, promoting protein folding and reducing protein translation by affecting mechanisms involved in protein synthesis and polypeptide formation (75, 83). This process increases ER-associated degradation (ERAD) and reduces protein translation by affecting mechanisms involved in protein synthesis and polypeptide formation (75). Within the ER, transmembrane sensors such as BiP, GRP78, and intraluminal chaperones are inactivated when they detect calcium depletion. The UPR is activated only after three transmembrane proteins, namely, inositol-requiring protein 1 (IRE1), dsRNA-activated protein kinase (PKR)-like ER kinase (PERK), and activating transcription factor 6 (ATF6), dissociate from BiP/GRP78, which serves as an inhibitory binding chaperone.

In healthy states, due to IRE1 activation, XBP1 is spliced (sXBP1) and plays a vital role in activating other ERAD pathway genes, which further halt protein translation. Furthermore, PERK initiates phosphorylation of elF2-α subunit and activates ATF4, and it helps in activating ERAD, amino acid metabolism, antioxidant stress response, CHOP, and GADD34 activation (84). ATF6 is activated in the Golgi and it functions as a transcription factor that promotes the expression of XBP1, CHOP, and ER chaperons. Among all of them, CHOP is crucial for ER associated apoptosis. In normal conditions, all the above-mentioned factors play key roles in maintaining homeostasis in cells; but in adverse conditions, their overexpression induces inflammatory response by activation of NFκB and IL-1β (74, 82). Additionally, the JNK pathway is activated by the ER stress-induced response, which is critical for the progression of this disease (84). ER stress-induced apoptosis is caused due to the upregulation of proapoptotic proteins like CHOP, cell cycle arrest, and DNA damage (gene/protein) such as GADD34/135. Caspase 12 and 4, JNK, and IFN3 signaling components are increased as a result of ER stress and cause cell death (85).

The lipid peroxidation products MDA, MAA, 4-HNE, and acrolein are highly active and can impair structure and activity, ultimately leading to the generation of ER stress. Smathers et al., (86), concluded in their study that 4-HNE contributes to modifying Hsp70, Hsp90, and protein disulfide isomerase (PDI) in an animal model. All these targeted proteins are important for ATPase activity, the rearrangement of misfolded protein, and dysfunctional restoration of mismatched impaired protein disulphide bonds. These are the mechanisms by which 4-HNE contributes to ER stress and hepatic lipid accumulation. Acrolein is also one of the lipid peroxidation byproducts, which is not well explored in alcoholic liver disease. Chen et al., (87), showed in their work that alcohol consumption leads to hepatic acrolein accumulation and a decrease in glutathione-s-transferase-Pi (GSTP) that helps to metabolize acrolein. Acrolein induces proapoptotic signals and increases in ER stress. Furthermore, adducts formed due to acetaldehyde bind to advanced glycation end products and increase ROS generation, hepatocyte ballooning, apoptosis, and steatosis by hepatic fatty degeneration and gut leakiness (88). Acetaldehyde also impairs mitochondrial glutathione and sensitize cells to TNF (89).

In addition to the aforementioned mechanisms, it has been demonstrated that ER stress contributes to both inflammation and lipogenesis. The presence of essential enzymes on ER would further renders the crucial site for lipid metabolism (90). ER stress genes, predominantly the PERK/ATF4 pathway, can activate SREBP1c, which in turn elevates the concentrations of FAS and ACC (91, 92). According to (93)‘s research, ER stress causes elevated levels of in lipogenic enzyme due to the cleavage of SREBP1C by site 1 protease (S1P) and site 2 protease (S2P). Many researchers describes ER stress-mediated disease progression as the second stage of alcohol-related liver disease as a result of upregulated ER stress inflammatory and steatosis pathways.

Prolonged ER stress promotes cell apoptosis by activating NFκB and inhibiting the translation of IκB, leading to macrophage activation generating an inflammatory response. Along with these other mechanisms, ER stress also influences the activation of lipogenic factors such SREBP1C (84, 94). Currently, it can be said that oxidative stress generated due to ethanol metabolism is an important factor for ALD progression. Researchers have also carried out experiments with zebrafish to prove this theory (95). As fat accumulation and inflammation increase in the liver, they can also disrupt the gut environment and contribute to disease progression.

## 5 Alcohol and its relationship with gut microbiota

Collectively, the bacteria, fungi, viruses, archaea, and eukarya colonizing the GI tract are termed ‘gut microbiota’ (96). The gut microbiota are vital components of our daily lives, actively contributing to vitamin synthesis, the creation of both essential and non-essential amino acids, and the production of short-chain fatty acids. They form a mutualistic relationship with the host and protect against environmental factors and some antigens by generating immunogenic responses (97). The gut contains more than 10^12^ to 10^14^ microbial cells, which is nearly equivalent to the total number of the cells in entire body (98). Scientists became interested in the mutualistic link between the host and gut microbiota because changes in gut bacteria are critical in the development of disease and also affect the metabolism of many vitamins, amino acids, enzymes, and short chain fatty acids. From infancy until adulthood, several bacterial alterations take place to maintain homeostasis.

### 5.1 Alcohol, gut dysbiosis, and their effect on gut flora

Alcohol does not only affect liver; it also affects other organs like the brain (99), gut intestinal track (100), kidney (101), breast (102), and heart (103). Due to excessive alcohol consumption, gut microflora also gets disturbed (104). According to (105), when an excessive amount of alcohol is administered, the established bacterial community will seek more alcohol because it will assist to maintain its dominance in the gut ecosystem. Additionally, they looked at the higher Actinobacterial concentration in the gut of the alcohol group. Firmicutes and Bacteroidetes made up the majority of the gut microbiota, while Proteobacteria, Verrucomicrobia, Actinobacteria, and Fusobacteria were also present but in the minority (106).

Firmicutes, a class of bacteria with a predominance of gram-positive bacteria, and Bacteroidetes, a class of bacteria with a predominance of gram-negative bacteria, are more prevalent in the gut microbiota in normal conditions, making up approximately 90% of it (96, 106). But, comparing Firmicutes and Bacteroidetes, the concentration of Firmicutes is higher. Other bacteria classes include Proteobacteria, Actinobacteria, Fusobacteria, and Verrucomicrobia (106–108). Firmicutes are composed of many bacteria like *Lactobacillus* and other opportunistic pathogenic bacteria like *Clostridium* which are relatively low. The Firmicutes and Bacteroidetes (F/B) ratio is important to maintain intestinal homeostasis. As the F/B ratio is increased, depending on lifestyle, food intake, and other environmental factors, gut homeostasis is affected. Due to a heavy alcohol intake, the F/B ratio gets disturbed and increases and, because of this, gut dysbiosis is generated (109). Additionally (110), mentioned that due to a high fat diet, Bacteroides and Actinobacteria are majorly benefited while the opposite goes for Firmicutes and Proteobacteria. This explains that diet and lifestyle affect gut microbial composition. Similarly, alcohol changes gut microbiota by enhancing the F/B ratio (111). showed that due to excessive alcohol consumption, Proteobacteria and many species of Bacteroidetes benefit but most abundant phyla of Firmicutes are inhibited in the presence of alcohol. Mainly, Lactobacillus and Bifidobacterium along with *Faecalibacterium prausnitzii* and *Akkermansia muciniphila* are inhibited (112). Such a shift from good bacteria to harmful bacteria results in gut dysbiosis and affect bacterial products. It further triggers other immune regulators and helps in disease progression.

The gut microbiota is composed of a mixture of bacteria, fungi, viruses, and archaea. While the bacterial component of the microbiota is well understood, other microbial groups have not been extensively studied. Nevertheless, they also play a significant role in maintaining eubiosis and promoting gut homeostasis (113). Ethanol consumption leads to fungal dysbiosis, which in turn contributes to the generation of immune responses (114). Common fungal species found in the gut include *Candida* spp., *Saccharomyces cerevisiae*, *Penicillium commune*, and *Aspergillus versicolor* (115). These species are present in higher concentrations, while others such as Galactomyces, Debaryomyces, Cladosporium, and Trichosporon are present in lower abundance. With increased alcohol consumption, the concentration of *Candida albicans*, a pathogenic fungus known to induce inflammatory responses, also increases (114). Conversely, the concentrations of Galactomyces, Debaryomyces, and *Saccharomyces cerevisiae* decrease (116, 117). Patients with alcoholic hepatitis have been found to exhibit reduced fungal diversity due to the overgrowth of *Candida albicans* (118). In patients with alcoholic hepatitis, the presence of anti-*Saccharomyces cerevisiae* antibodies indicates a systemic immune response against fungal products (116).

Gut viruses are different in every person and because of that are recognized as virus fingerprints. Some scientists describe them as ‘dark matter’ in the intestine. The human gut contains 10 times more phages than symbiotic bacteria. According to GVD, 97.7% of the viral population are phages, 2.1% are eukaryotic viruses, and the remaining are archaeal viruses (119). In normal conditions, the majority of phages come from the *Caudovirales* order. In chronic ethanol feeding, gut virome abundance is also disrupted and the concentration of Lactobacillus phages and Propionibacterium phages decrease while Streptococcus and Lactococcus phages are increased and help in the progression of disease severity (120, 121).

### 5.2 Consequences of gut dysbiosis due to alcohol

The shift from good to bad bacteria disrupts many intestinal mechanisms such as the production of many short chain fatty acids (SCFA) like Butyrate, change in the mucus layer integrity, endotoxemia, increased bacterial acetaldehyde dehydrogenase activity, and intestinal impermeability development. Due to all these conditions, gut leakiness can occur. In every step, molecular mechanisms are involved which are directly or indirectly related to the gut dysbiosis generated due to heavy alcohol intake (122, 123).

- Gut microbes help to ferment indigested dietary products into SCFAs. Due to alcohol consumption, this process is negatively impacted in the conversion of Butyrate co-A to Butyrate. It occurs due to change in 2 butyryl-CoA: acetate CoA transferase (BUT) and the butyrate kinase (BUK) gene (124). The impact of ethanol on gut tight junctions and gut metabolites is still unknown. Researchers have shown interest in decoding these particular mechanisms in order to discover how ethanol changes intestinal metabolite composition and how those metabolites protect against ethanol driven injury. Several researchers have explored the positive impact of exogenously administered SCFAs, namely butyrate, propionate, and acetate. These compounds appear to enhance AMPK activity and reduce metabolic stress. Even the tight junctions of Caco2 monolayer cells are restored after treatment with SCFAs (125–128). SCFAs are further helpful in generating immune signal cascades coupled with G protein (129). CPT1A gene expression is important for fatty acid oxidation. Due to a heavy alcohol intake, CPT1A expression decreases. But when the butyric treatment is given, it acts as an inhibitor to histone deacetylase (HDAC) and increases CPT1A expression (130). In this way, alcohol consumption changes butyric concentrations, which is one of the essential SCFAs.
- Muc2 overexpression is observed in alcoholics, which further correlates with antibiotic resistance and gut leakiness (permeability). Due to gut dysbiosis, Muc2 expression is increased which further acts as a glycan source for the bacteria and the overgrowth of undesirable bacteria is observed. In many muc2 deficient mice, alcoholic steatohepatitis is delayed (131–135). But there are still some contradictory studies that deny these observations. Therefore, further studies still need to be performed in order to understand the mechanism.
- Gut leakiness is generated due to ethanol which affects the tight junctions of intestinal cells, which leads to alterations of gut permeability. Increased acetaldehyde concentrations brought on by bacterial alcohol dehydrogenase activity causes leaky gut (136). Tight junctions and adherent junction protein’s integrity mainly depend on protein phosphorylation and dephosphorylation. Many proteins are involved in tight junction integrity. Acetaldehyde is one of the major effective molecules that rearranges the components of tight junction proteins. Due to increase in acetaldehyde, tyrosine phosphorylation of ZO-1, E cadherin also increases. This phosphorylation activity can be attenuated via protein tyrosine phosphatase activity which is hindered because of the increased concentration of acetaldehyde (137, 138). Further endotoxins trigger the dysregulation of tight junction proteins by activating NFκB, TNF-α mediated damage, and the downregulation of ZO-1 (139, 140).
- Zinc is a trace element that helps to maintain cell homeostasis, detoxification, antioxidant defense, and in gene regulation. Zinc acts as a nutraceutical substance to maintain the barrier function of the epithelium (141). Zinc deficiency is another factor for gut permeability by disassembling tight junction proteins (142), and, due to zinc deficiency, antioxidant mechanisms also get disrupted and inflammatory response gets activated (143–146). It is also associated with hepatic nuclear factor 4α (HNF4α) activity and causes damage to lipid metabolism by dysregulating PPARα (147–149). Zinc helps to maintain the intestinal barrier integrity and regeneration of impaired epithelium. It helps in invading molecular ions and pathogens by occluding proteolysis and occluding transcription. By barrier disfunction, neutrophil infiltration will be carried out and mucosal inflammation will be carried away (150, 151).
- Reg3 gene encodes a number of regenerating islet-derived genes which show antimicrobial and bactericidal activity (109, 152, 153). This family of genes plays an important role in maintaining gut homeostasis and acts as an antimicrobial defense mechanism (154, 155). Chronic ethanol feeding reduces the expression of Reg3 in the intestine (156–158). This compromised mechanism further contributes to disease progression.

There are still different mechanisms which need to be uncovered and found to understand the mechanisms behind disease progression so that some therapeutic targets can be generated which can help in disease amelioration. For instance, Reg3 protein genes have antimicrobial and bactericidal activity and are connected to the MYD88 pathway but the cell signaling behind these still needs to be explored (159). Further, gut dysbiosis due to ethanol dysregulates indole 2 acetic acid (IAA), type 3 innate lymphoid cells, and aryl hydrocarbon receptors (AHR), and causes an increase in inflammation (156).

### 5.3 Inflammation due to gut dysbiosis

As gut dysbiosis takes place, endotoxin and other material from the gut first enter the liver via the portal vein. In normal conditions, liver sinusoidal endothelial cells (LSEC) help to maintain homeostasis by removing toxicants, viruses, waste products, and lipopolysaccharide (LPS). They also play a key role in maintaining quiescent hepatic stellate cell conditions. But in adverse conditions, they convert to their proinflammatory phenotype and further also help in activating quiescent hepatic stellate cells. LSEC produce proinflammatory cytokines like TNF-α, MCP-1, and macrophage inflammatory protein -1 (MIP-1). They also generate immune response and activate Kupffer cells and HSC.

Hepatic stellate cell activation is a significant feature in the progression of alcoholic liver disease. These cells, known for their role in lipid storage, particularly retinoic acid (Vitamin A), are impacted by increased alcohol intake. The activity of CYP2E1 interferes with retinyl ester formation and disrupts the balance of the extracellular matrix (160, 161). Furthermore, bacterial lipopolysaccharides (LPS) and endotoxins not only activate Kupffer cells but also stimulate hepatic stellate cells (162, 163). This activation triggers the MyD88-dependent pathway, leading to the production of inflammatory cytokines and transforming growth factor β (164). Activated Kupffer cells produce interferon regulatory factors, which in turn activate quiescent hepatic stellate cells, transforming them into myofibroblasts (165). Pit cells, also referred to as natural killer (NK) cells, normally exhibit antifibrotic and anti-inflammatory activities. However, in diseased conditions, their functionality is compromised, leading to an inability to carry out their intended functions (114). Additionally, their cytotoxicity towards activated hepatic stellate cells is reduced due to changes in their surface ligands and the accumulation of higher levels of TGF-β (166).

The interaction between the gut and liver plays an essential role. Liver residential macrophages- Kupffer cells play a significant role. There are two types of Kupffer cells, M1 and M2, and they switch from M2 to M1 as per immunogenic conditions. M1 is activated because of bacterial products like lipopolysaccharides (LPS) and produces proinflammatory molecules. The moment when M1 is activated, M2 macrophages, which are mainly known for anti-inflammatory activity, are suppressed (167, 168). As mentioned earlier, ethanol and its byproducts create gut dysbiosis, causing endotoxemia. As the release of LPS endotoxins increase, they can enter the blood circulation system and hence can travel to many organs. It can also cross the blood–brain barrier and liver parenchymal layer, causing maximum damage. Endotoxins travel through the portal vein and enter the liver. Due to LPS, liver Kupffer cells are activated via pathogen-associated molecular patterns (PAMPs); Tall like receptor (TLR4). LPS-binding protein (LPB) helps LPS to translocate and bind to CD14. It further facilitates binding to the TLR4/MD2 complex. These endotoxins or LPS bind to TLR4 in order to support MD2 and CD4+. This complex further targets two major mechanisms. The reason behind the triggering of specific mechanisms is still a gray area. Majorly, the MyD88 and TRIF pathways are activated. Both the pathways produce different types of inflammatory molecules and further cause more liver injury (169–173). Kupffer cell activation mechanisms are well known and have been extensively studied, but the activation resulting from fungal expression is not as well investigated. The C-type lectin-like receptor (CLEC7A) on Kupffer cells has the ability to bind to 1-3β glucan, which is present on the fungal cell wall. Upon activation, Kupffer cells produce IL-1β (174).

## 6 Treatment for alcoholic liver disease

Alcohol damages not only the liver but also affects other organs and disrupts their physiological mechanisms. If the right abstinence conditions, diet, and healthy lifestyle are adopted, ALD can be treated in its early stages and reversed. There are some targeted drugs that are being observed in clinical trials. S-adenosyl methionine and granulocyte colony stimulation factor drugs produced average results in clinical phase 2 trials (175). Furthermore, there are other pharmaceutical agents are being studied clinically and, from them, metadoxine completed a phase IV trial and produced effective results for severe alcoholic hepatitis (176). There are several other steroid-like corticosteroid-based treatments available, but if positive results are not observed then they are discontinued. Other anti-inflammatory treatments, such as pentoxifylline (15), antioxidant treatments, like SOD, and natural antioxidant treatments, like silymarin (33), are also available. Research is ongoing for probiotics (177–179) and synbiotics (180) as well. VSL#3 is in clinical trials (Clinical.Trail.gov identifier no. NCT05007470). In the end stage of liver disease, liver transplantation is the only option and even after liver transplantation, proper lifestyle and diet changes should be maintained.

### 6.1 Nanoparticles and their use as a therapeutic agent

Though there are various treatments available, none are FDA approved and have some drawbacks. To minimalize the drawbacks, nanoparticles are used. There are various type of nanoparticle and nano-formulations are used to check against different types of liver disease, targeting the same mechanism involved in ALD. The use of nanoparticles for ALD should be studied more. Some of the research done on various liver diseases and nano-formulations are mentioned in **Table 1**. There are several bare nanoparticles used to treat liver disease mentioned in **Table 2**.

**Table 1:** Nano-formulations and their role in liver disease.

Sr no.	Particle formulation	Original drawback	Nano-formulation	Targeting mechanism	Disease relevant	Reference
1	Rapamycin	Water insoluble	mPEG-PLGA	Decrease triglyceride accumulation, decreased SREBP1c-mediated lipid generation, and increased PPAR-α-mediated fat oxidation	NAFLD *In vivo* and *in vitro* study	(181)
2	Resveratrol	Water insoluble and Poor intestinal metabolism	PLGA	Alleviated lipogenesis and lipolysis, and reduced hepatocellular proliferation compared with free resveratrol.	NAFLD *In vivo* and *in vitro* study	(182)
3	Curcumin	Water insoluble	Nano curcumin		NAFLD Human study (placebo-controlled clinical trial)	(183)
4	Silymarin	Water insoluble and low bootability	Gold nanoparticle	Enhanced antifibrotic activity	CCl_4_-induced hepatic fibrosis	(184)
5	Silymarin	Water insoluble, low oral bioavailability, low membrane permeability, and low bootability	Chitosan-lipid polymer hybrid	Higher uptake by fatty liver cell, reduced triglyceride lipid, lowering effect, and enhanced oral delivery.	Transgenic NAFLD model	(185)
6	Phyllanthin	Low water solubility	PLGA	Reduced liver marker enzyme and collagen levels.	CCl4-induced hepatic fibrosis. Toxicity study on zebra fish embryos	(186)
7	Naringenin	Poor water solubility, low oral bioavailability	Nanostructured lipid carrier	Increased solubility, stability, intestinal absorption, and bioavailability	NAFLD (methionine choline diet)	(187)
8	Roselle Seed Oil (*Hibiscus sabdariffa L.*)	Poor water solubility	Nanoemulsion using Tween 80 and Polyethylene glyco (PEG).	Increased GSH and NrF2 and decreased MDA (reduced oxidative stress and proinflammatory cytokines)	Paracetamol intoxication in liver	(188)
9	Luteolin	Low water soluble and low bioavailability	Zinc oxide nanoparticle	P13K/AKT/FoxO1 signaling pathway. Improved hepatic insulin sensitivity, anti-oxidant, and lipid lowering effect	Non alcoholic steatohepatitis (NASH) and diabetic rat model	(189)
10	SOD1	–	Cu/Zn Poly L lysine- polyethylene glycol copolymer (Cu/Zn SOD PLL PEG)	Reduced hepatic oxidative stress, increased PPAR-α mediated fatty acid oxidation, and increased adipose tissue lipolysis	NASH/ALD mice model	(190)
11	CoEnzyme Q10	Lipid solubility, low bioavailability, and higher dosage for oral administration	Coenzyme Q10 nanoparticle	Antioxidant properties, free radical scavenging properties	Dichlorvos-induced hepatic toxicity in adult male Wistar rats	(191)
12	Silibinin (SBN)	High dosage for longer period of time administration	PLGA	Enhanced expression of phase II enzyme, antioxidant mechanism with single shot of SBN-PLGA nanoparticles	Dacarbazine-induced hepatic toxicity	(192)

**Table 2:** Ameliorating effect of nanoparticles on liver disease.

Sr. no.	Nanoparticle	Targeting mechanism	Disease condition and model	Reference
1	Cerium oxide nanoparticle	Antioxidant mechanism with decreased peroxidation markers	NAFLD with fipronil insecticide on male albino mice	(193)
4	C60(OH)10/2-Hydroxypropyl-β-cyclodextrin	Antioxidant	HeLa and HepG2 cells against H_2_O_2_-mediated cell death	(194)
5	Ginger-derived nanoparticle	Activation of NrF2-dependent antioxidant pathway	ALD mice model	(195)
6	Spherical neutral gold nanoparticle	Downregulating the activity of Kupffer cells and hepatic stellate cells, fibrosis and oxidative stress by modulating AKT/PI3K and MAPK signaling pathways	ALD mice model	(196)
7	Biosynthesized nano silver from *I. oblongifolia leaves*	Antioxidant, anti-apoptotic, and anti-inflammatory	Liver damage caused due to blood-stage malaria	(197)
8	Zinc oxide	Decreased expression of SREBP1c, FAS, and ACC. Stabilized SIRT1 expression.	NAFLD mice	(198)
9	Cerium oxide nanoparticle	Scavenger of ROS, acts as anti-oxidant, and improves hepatic proliferation and liver regeneration	Acetaminophen-induced liver injury	(199)
10	Cerium oxide nanoparticle	Antilipogenic effect	*In vivo* NAFLD model	(200)
11	Selenium oxide nanoparticle	Increase expression of Seleno protein in ApoE-deficient mice and reduce oxidative stress	ApoE-deficient mice	(201)
12	Cerium oxide	Increased total antioxidant capacity (TAC), glutathione. Lowered TNFα, MDA, and total oxidative status (TOS). Histological structures were maintained due to Nanoparticle (NP) treatment	NAFLD and CCl_4_-induced liver and intestine damage	(202)
13	Citrate functionalized Mn_3_O_4_	Increased antioxidant activity	*In vivo* CCl4-induced hepatic fibrosis and cirrhosis	(203)
14	Silver nanoparticle (Green synthesis)	Antioxidant mechanism and hepatoprotective	Paracetamol-induced toxicity	(204)
15	Gold nanoparticle	Antioxidant and hepatoprotective effect, free radical scavenging property	Murine hepatic schistosomiasis	(205)

Moreover, to normalize gut microflora, nanoparticle-mediated probiotic treatment has also recently been studied by many researchers. Most of them are mentioned in **Table 3**.

**Table 3:** Probiotic nanoparticles for intestinal gut dysbiosis.

Sr. no.	Probiotic strain and nanoparticle	Targeting mechanism	Diseased condition and model	Reference
1	*Bacillus amyloliquefaciens* and chitosan nanoparticle	Decreased oxidative stress, inflammation in colon, and maintained microbial population	Mice model- dextran sulfate sodium salt (DSS)-induced colitis	(206)
2	*Lactobacillus GG* derived exosomes like nanoparticle	Reduced LPS inflammation, increased aryl hydrocarbon receptor, increased tight junction protein. Decreased hepatic bacterial translocation	ALD model *In vitro:* RAW264.7 *In vivo:* C57BL/6J mice	(207)
3	Lactobacillus and Bifidobacterium	Reversed oxidative DNA damage, reduced MDA, and advanced glycation end product	Cadmium (Cd)-derived toxicity in male Wistar rats	(208)
4	Selenium nanoparticle by *Lactobacillus casei*	Maintained intestinal epithelial integrity, intestinal microflora balance, and ROS	Mice model with enterotoxigenic *Escherichia coli K88* *In vitro:* NCM460, IPEC-J2, HepG2, and THP-1 cell lines used for toxicology study.	(209)

## 7 Conclusion

Excessive alcohol consumption is a lifestyle-related issue that poses significant harm to various organs, with the liver being one of the most affected. It disrupts the balance of reactive oxygen species (ROS) and antioxidant mechanisms, leading to an imbalance in liver homeostasis. Alcohol also contributes to gut dysbiosis, further exacerbating the progression of the disease towards irreversible stages. While several drugs have been developed for the treatment of alcoholic liver disease (ALD), none have received FDA approval as targeted therapies. There are numerous underlying mechanisms involved in ALD, many of which remain unexplored. Recently, researchers have shown interest in nanomedicine as a potential targeted treatment approach. Nanoparticles are being investigated for various chronic liver conditions due to their ability to minimize side effects, deliver drugs specifically to the intended site, and enhance the bioavailability of natural compounds. Some nanoparticles have shown promising results by targeting the same mechanisms involved in ALD. Although finding a targeted drug for ALD is crucial, it is equally important to uncover other mechanisms implicated in the progression of the disease.

## Author contributions

HA and DZ: conceptualization. PC and DZ: formal analysis and investigation. HA: writing—original draft preparation. PC and DZ: writing—review and editing.PM: supervision. All authors contributed to the article and approved the submitted version.
